# Adult hamartoma of the chest wall: A case report

**DOI:** 10.3389/fonc.2022.1004837

**Published:** 2022-10-27

**Authors:** Dong Bai, Yan-han Liu

**Affiliations:** Department of Radiology, Aerospace Center Hospital, Beijing, China

**Keywords:** hamartoma, chest wall, soft tissue tumor, CT, MRI

## Abstract

This paper retrospectively analyzed the case data of an adult hamartoma of the left chest wall, and combined with the literature analysis, to discuss the clinical characteristics, imaging diagnosis and differential diagnosis of hamartoma. CT and MRI findings of the patient showed medial occupation of the left serratius anterior muscle, with visible fat and calcification. Different from the previous reports of chest wall hamartoma in terms of age, location and imaging characteristics, this case had certain characteristics, and the diagnosis needed pathological confirmation after surgical resection.

## Introduction

The word hamartoma comes from the Greek “hamartanein”, meaning to go wrong, and was introduced by AlBrecht in 1904 (1). It is characterized by the formation of a tumor-like mass, which consists of localized, overgrown, inherent mature tissue in the wrong proportion, without the normal arrangement of tissue structure (2). It may occur in any organ. Domestic and foreign literature reports on chest wall hamartoma mainly focus on infants with ribs as the origin, but hamartoma occurring in adults without ribs is quite rare and the number of reported cases is limited. Here we introduce a case of chest wall hamartoma in a middle-aged female, and discuss the characteristics, diagnosis, differential diagnosis and treatment of the case. Written informed consent was obtained from the individual for the publication of any potentially identifiable images or data included in this article.

## Clinical data

A 59-year-old female patient was admitted with the chief complaint of “left shoulder mass for 4 years”. The patient underwent chest CT examination in our hospital 4 years ago and showed soft tissue occupation in the left anterior shoulder blade. The patient did not have chest distress, chest pain, palpitation, shortness of breath, chest wall skin swelling and rupture, fever, chills and other discomfort, and no special treatment was given. After that, the patient underwent regular physical examination every year, and the left scapular mass was found to increase slowly. Half a month ago, the patient underwent physical examination again in our hospital, and chest CT showed soft tissue mass in the left anterior scapula with calcified nodules, suggesting further examination. He was admitted for further treatment on August 26, 2021.Physical examination showed that the chest was symmetrical without deformity, the left posterior back was protruded, the texture was hard, there was no tenderness, the skin was not redness, swelling and ulceration, the bilateral respiratory movement was consistent, the bilateral tactile chatter was symmetrical, the bilateral lung percussion sound was clear, the bilateral lung breathing sound was clear, no dry and wet rale was heard, and no pleural friction. The patient had previously received cervical polypectomy in our hospital. Hyperlipidemia was treated with rosuvastatin orally for 5 years.She suffered from depression for 10 years. No infectious or chronic disease or history of trauma was reported.

**Figure 1:**
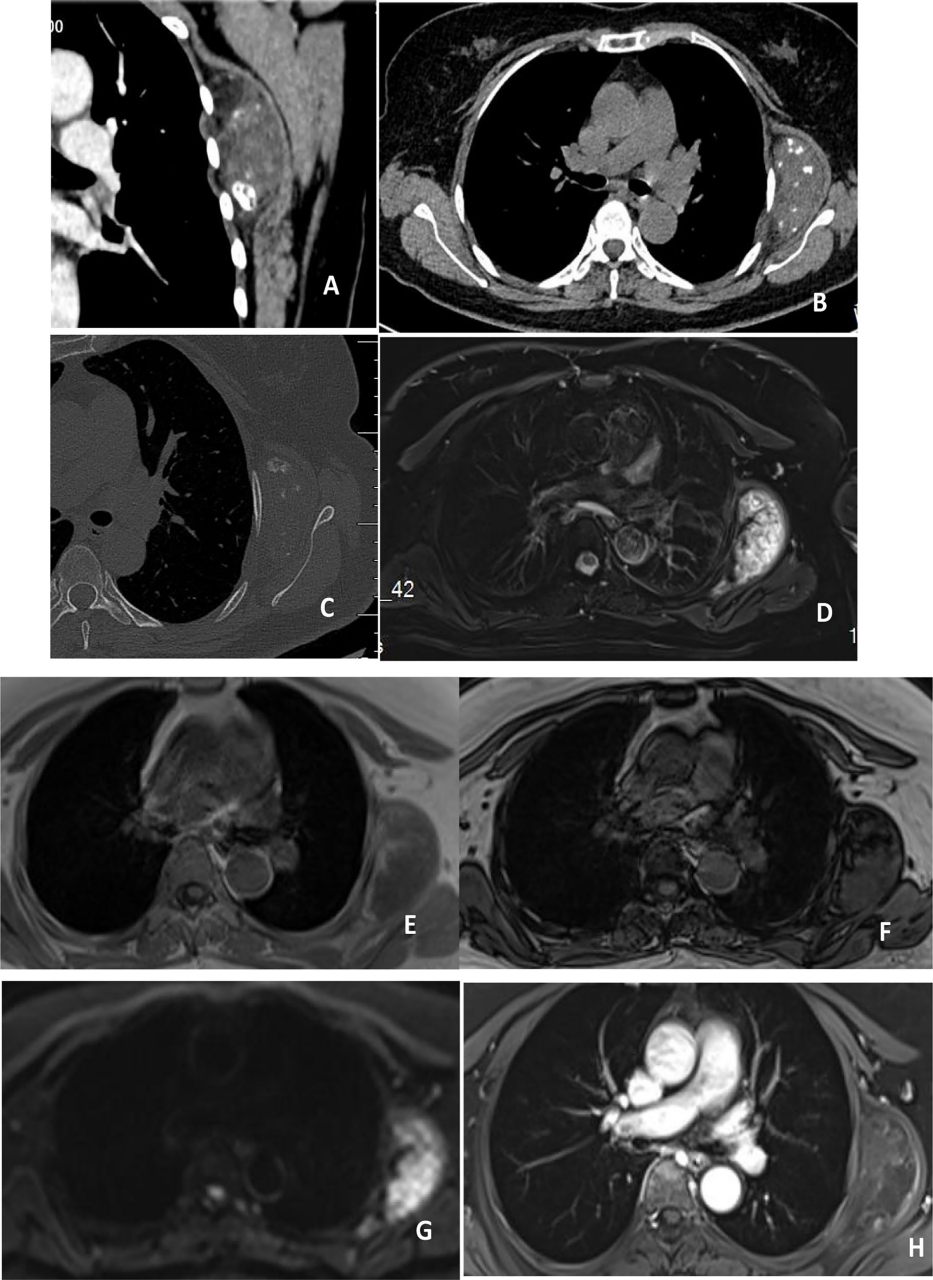
**(A–C)** showed plain and enhanced CT scan, showing mixed density foci inside the left serratus anterior muscle, calcification and fat density, no significant enhancement after enhancement, and no bone destruction in adjacent ribs. **(D–H)** showed plain and enhanced MRI, with high signal on T2WI and stripe low signal inside the left serratus anterior muscle, isosignal in T1WI, stripe high signal inside, low signal in reverse phase, and high signal in DWI. After enhancement, no significant enhancement was observed in the center of the lesion, and enhancement was observed at the edge.

Chest CT (**Figure 1**) showed a semicircle mixed density shadow under the muscularlayer in front of the scapula on the left side of the chest wall, multiple nodular calcification foci and fat density foci were observed in the chest wall, with a clear boundary and a size of 63.2*35.3*64.2mm. Enhancement was not obvious on enhanced scan. MRI (**Figure 1**) of the chest showed a mass between the left serratus anterior and the ribs, which was considered as a benign lesion. Elastofibroma might be large.

**Figure 2:**
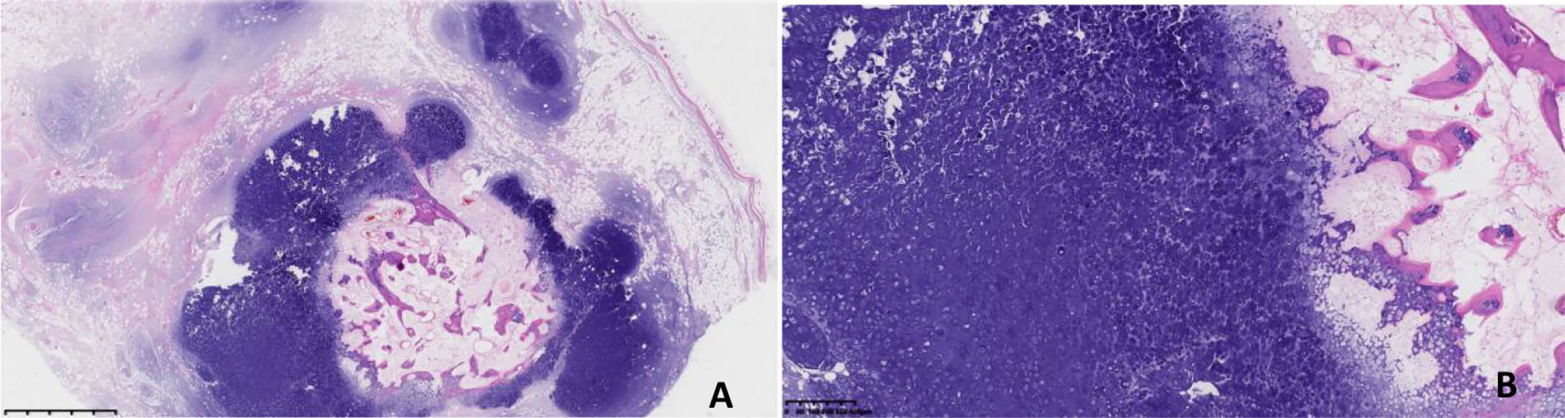
(**A**, HE×10; **B**, HE×40) Under the light microscope, the tumor showed multi-nodular growth, fat wrapped around the nodular growth cartilage, calcification in the center of some cartilage nodules, and no obvious atypia of chondrocytes.

The patient completed preoperative examination on August 31, 2021 under general anesthesia, “left thoracic wall soft tissue tumor resection + negative pressure suction implantation”. After the patient was admitted to surgery, intraoperative ultrasound examination was performed to determine the location of the tumor, and the skin was cut about 4cm above the left thoracic wall tumor in the armpit to separate the subcutaneous tissue and muscle. There was a mass on the deep surface of serratus anterior muscle with regular shape, clear boundary and good range of motion. The tumor was removed completely along the blunt + sharp surrounding layer by layer. Postoperative histopathological examination (**Figure 2**) revealed a nodular mass (on the left shoulder and back) with a size of 7.5x6x3.5cm and complete capsule. The section was gray and yellow, hard and decalcified. (left shoulder and back) examination showed nodular growth of mature cartilage, focal ossification, consistent with hamartoma. Postoperative chest CT reexamination of the patient on October 19, 2021 showed no clear signs of recurrence.

## Discussion

The word hamartoma comes from the Greek “hamartanein”, meaning to go wrong, and was introduced by AlBrecht in 1904 (1). It is characterized by the formation of a tumor-like mass, which consists of localized, overgrown, inherent mature tissue in the wrong proportion, without the normal arrangement of tissue structure (2). Hamartomas are not real tumors. They don’t metastasize. Although it may occur in any organ, such as lung, kidney, liver and spleen, hypothalamus, perineum, throat, etc.Domestic and foreign literature reports on chest wall hamartoma mainly focus on infants with ribs as their origin, but hamartoma occurring in adult chest wall without involving ribs is quite rare and the number of reported cases is limited (3, 4). There is no significant difference in the incidence rate of chest wall hamartoma between males and females in most cases, and it is more common in infants and infants than in adults. Most adult cases are clinically detected by physical examination, and they are clinically asymptomatic when they are small in size. Non-specific symptoms, such as chest pain and cough, can be caused by a large mass pressing on surrounding tissues.In this case, a middle-aged woman was found by physical examination without obvious clinical symptoms.

Pathologically, hamartoma can be divided into two types: mesenchymal hamartoma and epithelial or glandular hamartoma (4). Mesenchymal hamartomas are disordered by overgrown mesoderm tissue without any epithelial component. In contrast, epithelial or glandular hamartomas consist of epithelial or glandular components mixed with mesodermal tissue without obvious atypia (5). In this case, the mature cartilage was of mesenchymal type, with fat wrapping around the cartilage and calcification in the center of some cartilage nodules. Chondrocytes had no obvious atypia and lack the ability of uncontrolled progressive growth. Generally, they will not develop into malignant tumors.

In terms of imaging, the imaging manifestations of chest wall hamartoma have certain specificity, mainly calcification and fat. CT is sensitive to calcification, and a large range of fat can also be found, but MR shows more clearly for lesions with less fat. MR is more advantageous than CT for the extent of lesions.In 18F-FDG PET-CT, hamartomas usually have no or only slight increase in uptake, and the uptake on delayed imaging is often unchanged or decreased. FDG PET-CT can better determine the benign and malignant lesions than MSCT (6). Sun Ping et al. (7) reported 2 cases of adult thoracic hamartoma with rib bone destruction, high and low mixed density, and multiple calcification density shadows. The mass protruded into the lung area, and the boundary between the mass and the lung was still clear. Lei Yyan et al. (8) reported that among 38 cases of thoracic hamartoma, 4 cases were from chest wall, and the imaging manifestations were mainly rib enlargement and bone destruction, with multiple calcification foci in the lesions. The composition of hamartoma is complex, and fat and calcification are the main components, so the imaging has certain specificity.

In this case,the patient in this case was an adult female who was found to grow slowly, and its clinical symptoms were similar to those of hamartomas in other parts of the body. CT showed oval mixed density lesions on the medial side of the serratus anterior muscle, calcification and fat density lesions could be seen inside, and no clear damage or morphological changes could be seen near the ribs of the lesions on the window of bone. MRI could more clearly identify the scope and composition of the lesions, The lesions showed high signal on T2WI and low signal in strips, isointensity in T1WI, high signal in strips on T1WI, low signal in reverse phase, high signal on DWI, no significant enhancement in the center of the lesion after enhancement, enhancement at the edge, and high signal on DWI. Internal fat is hypointense in the fat-suppressed phase, which is a specific sign of hamartoma. The main differential diagnosis includes teratoma, elastic fibroma and neurogenic tumor. Teratoma is a germ cell tumor derived from primordial germ cells or pluripotent embryonic stem cells, mostly located in the midline or parastinal area of the body. Teratoma of the chest is more likely to occur in the mediastinum, and its imaging features are cystic and fatty, with calcification visible in 50%. The “fat-liquid plane” has more imaging characteristics, so it can be distinguished from chest wall hamartoma in terms of location and imaging characteristics. The location of hamartoma in this case is similar to that of elastic fibroma, which usually occurs in the deep subscapular horn, but the signal of elastic fibroma is similar to muscle tissue due to the large amount of collagen fibers in the tumor. There are fat components included in the tumor, and the imaging manifestation is “lipid striation sign”, but calcification is rare, and the enhancement is not obvious after enhancement. Neurogenic tumors in the intercostals nerve sheath tumor is relatively common, characterized by round or class round soft tissue density with more uneven density, border and clear, the uneven enhancement scan in mild-to-moderate reinforcement, for the larger part of the tumor, change, the necrotic area can see sac tumors had center, no obvious fat component and calcification is rare, therefore with hamartoma.

In terms of treatment, hamartoma is a benign tumor with small lesion in biology.Occurs in chest wall hamartomas and is treated in a similar manner to other sites. When there is no obvious symptom in clinic, observation should be the main method. If the lesion is too large to compress the surrounding tissue and cause clinical symptoms, complete surgical resection of the mass can be cured.

In conclusion, we report a rare case of thoracic hamartoma in an adult. Although hamartoma may occur in any organ, hamartoma occurring in chest wall without involving ribs is quite rare, and there are few reports in domestic and foreign literature. This article aims to summarize the clinical manifestations, pathological features, imaging findings and differential diagnosis for the mutual learning of the same way.

## Data availability statement

The original contributions presented in the study are included in the article/supplementary material. Further inquiries can be directed to the corresponding author.

## Ethics statement

This study involving a human participant was reviewed and approved by the ethics Committee of the Aerospace Central Hospital. The patient provided her written informed consent to participate in this study. Written informed consent was obtained from the individual for the publication of any potentially identifiable images or data included in this article.

## Author contributions

All authors contributed to the article and approved the submitted version.

## Conflict of interest

The authors declare that the research was conducted in the absence of any commercial or financial relationships that could be construed as a potential conflict of interest.

## Publisher’s note

All claims expressed in this article are solely those of the authors and do not necessarily represent those of their affiliated organizations, or those of the publisher, the editors and the reviewers. Any product that may be evaluated in this article, or claim that may be made by its manufacturer, is not guaranteed or endorsed by the publisher.

## References

[1] Albrecht E . Uber hamartoma. Verh Dtsch Geo Pathol (1904) 7:153–7.

[2] Leoncini G Maio V Mirabile L Baggi R Franchi A . Glandular hamartoma of the larynx: report of a case. ANL (2008) 35:149–51. doi: 10.1016/j.anl.2007.03.015 17851000

[3] Xing G Guo DA Lian Z . Pathophysiology and imaging of hamartoma. J Med Rev (2011) 17(6):937–9. doi: 10.3969/j.issn.1006-2084.2011.06.049

[4] Ahmed S Afreen SS Parthiban SR Anand HK Fatima Q Chavan P . A rare case of laryngeal hamartoma. Indian J Otolaryngol Head Neck Surg (2021) 73:1–3. doi: 10.1007/s12070-021-02498-9 PMC9895685 36742732

[5] Caltabiano R Cocuzza S Maira S Gurrera A Serra A Lanzafame S . Respiratory epithelial adenomatoid hamartoma:case report and literature review. Pathologica (2008) 100:185–8. 18841825

[6] Liu Y Wu N Zheng R Ying L Zhang WJ Zhang H . Positron emission computed tomography-CT manifestations of pulmonary hamartoma. Chin J Radiol (2013) 47(6):513–6. doi: 10.3760/cma.j.issn.1005-1201.2013.06.007

[7] Sun P Cai Y Lv H Xiao Y Gu YL . Clinicopathological observation of 2 cases of adult chest wall chondromal hamartoma. J Clin Pathol (2017) 24(10):742–6. doi: 10.3969/j.issn.1007-8096.2017.10.006

[8] Lei YY Luo HH Chen ZG Su CH Kuang JY . Clinical treatment of 38 cases of thoracic hamartoma. Guangdong Med J (2007) 28(5):746–7. doi: 10.3969/j.issn.1001-9448.2007.05.029

